# Impact of the detection of ζ-globin chains and hemoglobin Bart’s using immunochromatographic strip tests for α^0^-thalassemia (--^SEA^) differential diagnosis

**DOI:** 10.1371/journal.pone.0223996

**Published:** 2019-10-29

**Authors:** Supansa Pata, Witida Laopajon, Matawee Pongpaiboon, Weeraya Thongkum, Nattapong Polpong, Thongperm Munkongdee, Kittiphong Paiboonsukwong, Suthat Fucharoen, Chatchai Tayapiwatana, Watchara Kasinrerk

**Affiliations:** 1 Division of Clinical Immunology, Department of Medical Technology, Faculty of Associated Medical Sciences, Chiang Mai University, Chiang Mai, Thailand; 2 Biomedical Technology Research Center, National Center for Genetic Engineering and Biotechnology, National Science and Technology Development Agency at the Faculty of Associated Medical Sciences, Chiang Mai University, Chiang Mai, Thailand; 3 Center of Biomolecular Therapy and Diagnostic, Faculty of Associated Medical Sciences, Chiang Mai University, Chiang Mai, Thailand; 4 Thalassemia Research Center, Institute of Molecular Biosciences, Mahidol University, Nakhon Pathom, Thailand; Imam Abdulrahman Bin Faisal University, SAUDI ARABIA

## Abstract

α^0^-Thalassemia is an inherited hematological disorder caused by the deletion of α-globin genes. The Southeast Asian deletion (--^SEA^) is the most common type of α^0^-thalassemia observed in Southeast Asian countries. Regarding WHO health policy, an effective α^0^-thalassemia screening strategy is needed to control new severe α-thalassemia cases. In this study, a monoclonal antibody panel was used to develop immunochromatographic (IC) strip tests for detecting the Hb Bart’s and ζ-globin chain. Among 195 samples, all α^0^-thalassemia traits (78 α^0^-thalassemia (--^SEA^) and 4 α^0^-thalassemia (--^THAI^)) had low MCV or MCH values. The sensitivity, specificity, PPV and NPV of the IC strip tests for ζ-globin and Hb Bart’s for screening α^0^-thalassemia (--^SEA^) within the low MCV or MCH samples were 100%, 65.2%, 90.7%, 100% and 96.2%, 47.8%, 86.6%, 78.6%, respectively. All 4 α^0^-thalassemia (--^THAI^) traits were negative for ζ-globin chains but positive for Hb Bart’s using the IC strip tests. These results led to a α^0^-thalassemia screening being proposed in which blood samples are first evaluated by MCV, MCH and Hb typing. Samples with high MCV and MCH values are excluded for the presence of the α^0^-thalassemia gene. Samples with low MCV or MCH values are assayed using the developed IC strip tests, where only samples testing positive are further assayed for α^0^-thalassemia by PCR. Patients with Hb H, EA Bart’s or EF Bart’s diseases do not need to use this IC strip assay. Thus, in this study, a simple and cost effective α^0^-thalassemia point of care test was developed.

## Introduction

α-Thalassemia is a genetic disorder caused by a defect in the α-globin gene [1, 2], the severe form of which (α^0^-thalassemia) is characterized by the deletion of both pairs of linked α-globin genes, whereas a single α-gene deletion is present in individuals with α^+^-thalassemia. Accordingly, couples who carry the α^0^-thalassemia trait have a 25% risk of hemoglobin (Hb) Bart’s hydrops fetalis in each pregnancy due to the absence of α-globin genes [3–5]. Hb Bart’s hydrops fetalis is the most severe type of thalassemia and causes fetuses die in utero. Their mothers also often suffer from several obstetric complications and must cope with the psychological burden of carrying a nonviable fetus to term [6, 7].

Currently, new cases of Hb Bart’s disease still occur and need to be prevented [2, 8]. Providing appropriate genetics counselling to individuals identified α-thalassemia can prevent severe thalassemia disease and reduce the spread of the α-thalassemia gene [9–12]. Polymerase chain reaction (PCR) is currently the most commonly used technique to diagnose α^0^-thalassemia [13–16]. However, this technique is not widely employed in routine laboratories of rural hospitals in resource-limited countries. Thus, the development of more cost effective and simplified techniques for identifying α^0^-thalassemia carriers are greatly needed for incorporation into the routine thalassemia screening programs of health promotion policies.

In Southeast Asian countries, the Southeast Asian (SEA) deletion (--^SEA^) is the most common α^0^-thalassemia genotype [2, 8, 11, 17, 18]. The minute amounts of Hb Bart’s and ζ-globin chains in red blood cells (RBCs) are especially observable in α^0^-thalassemia subjects, including those with α^0^-thalassemia (--^SEA^) [19–24]. Using a monoclonal antibody (mAb) generated in our lab, we previously developed an immunochromatographic (IC) strip test for detecting Hb Bart’s in RBC hemolysates [21, 25–27]. In this study, using a panel of our generated anti-ζ-globin chain mAbs [28], we established another IC strip test that can detect ζ-globin chains in RBC lysates. The IC strips for Hb Bart’s and ζ-globin chain detection were affirmed for their potential use in α^0^-thalassemia differentiation, especially in α^0^-thalassemia (--^SEA^) carriers. The clinical sensitivity, clinical specificity, positive predictive value (PPV) and negative predictive value (NPV) of both IC strip tests were validated, and a new α^0^-thalassemia screening strategy was also proposed.

## Materials and methods

### Antibodies and reagents

The anti-ζ-globin chain mAbs PL2 (IgG1 isotype) and PL3 (IgG1) [28] and the mouse anti-Ag85B mAb clone AM85B-8B (IgG1) [29] were generated in our laboratory. Goat anti-mouse IgG antibody was obtained from Jackson ImmunoResearch (West Grove, PA, USA). EZ-Link^™^ Sulfo-NHS-LC-Biotin was purchased from Pierce (Rockford, IL, USA). Horseradish peroxidase (HRP)-labeled streptavidin and 3,3’,5,5’-tetramethylbenzidine (TMB) substrate were purchased from Invitrogen (Camarillo, CA, USA). Goat anti-mouse immunoglobulins antibody was obtained from KPL (Gaithersburg, MD, USA). The IC strip test for the determination of Hb Bart’s in RBC hemolysates was purchased from i+Med Laboratories Co., Ltd. (Bangkok, Thailand).

### Identification of an anti-ζ-globin chain mAb pair for use in an immunochromatographic strip test

To identify an anti-ζ-globin chain mAb pair suitable for use in an IC strip test, a sandwich ELISA was employed. The anti-ζ-globin chain mAbs PL2 and PL3 or the isotype-matched control mAb were first biotinylated using EZ-Link^™^ Sulfo-NHS-LC-Biotin according to manufacturer instructions. For the sandwich ELISA, the anti-ζ-globin chain mAbs PL2 and PL3 or the isotype-matched control (10 μg/ml) were coated on 96-well ELISA plates (Costar, Corning, NY, USA) in carbonate/bicarbonate coating buffer pH 9.6 overnight at 4°C. After washing, the plate was blocked with 2% skim milk in PBS at 37°C for 1 hour. Hemolysates of Hb Bart’s hydrops fetalis were added and incubated at 37°C for 1 hour. After washing, biotinylated anti-ζ-globin mAbs PL2 or PL3 or the isotype-matched control mAb at 10 μg/ml were added and incubated at 37°C for 1 hour. Subsequently, the antigen-antibody complex was detected by adding HRP-labeled streptavidin at 37°C for 1 hour. Thereafter, TMB substrate was added and the reaction was stopped using 1 N HCl. The absorbance was measured at O.D. 450 nm.

### Preparation of an immunochromatographic strip test for the identification of ζ-globin chains

The IC test strip tests were constructed using the generated anti-ζ-globin chain mAbs PL2 and PL3 [28] as described elsewhere [21]. The IC test strip consists of four components: a sample application pad, a conjugate pad, an analytical nitrocellulose membrane and an absorbent pad. The anti-ζ-globin mAb PL3-colloidal gold conjugate was prepared as previously described [21] and sprayed onto the conjugate pad at a spraying rate of 0.38 μl/mm. The nitrocellulose membrane was divided into two zones: the test line zone (T) and the control line zone (C). The test and control lines were formed by the immobilized anti-ζ globin chain mAb PL2 at 3 mg/ml with a spraying rate of 0.08 μl/mm and goat anti-mouse immunoglobulins at 1 mg/ml using the same spraying rate at 1 μl/mm for each line. Subsequently, the sprayed conjugate pad and jetted membrane were incubated for 4 hours at 37°C and then dried in a desiccator. After drying, the components of the strip test were assembled and then cut into individual strips (4.0 mm/strip).

### Determination of Hb Bart’s and ζ-globin chains using immunochromatographic strip tests

An EDTA-blood sample (100 μl) was diluted with RBC lysis buffer (1% Triton X-100 in PBS) in a 96-well plate to obtain 1:2 for Hb Bart’s testing and 1:200 for ζ-globin chain testing. The IC strips (either for Hb Bart’s or for ζ-globin chains), with an arrow pointing toward the sample well, were then vertically immersed in the hemolyzed blood for 5 minutes. The strips were then washed using washing buffer (0.05% Tween-PBS) until the background was clear. Subsequently, the reactive bands on the strips were read visually. For a positive result, 2 red-purple color bands appeared, one at the test line zone and one at the control line zone. For a negative result, only 1 red-purple color band was observed at the control line zone.

### Blood samples

One hundred ninety-five routinely leftover blood samples were used in this study. These blood samples were collected from various types of thalassemic patients, thalassemia carriers and healthy subjects using EDTA as anticoagulant. Hematologic data were determined using an automatic blood cell counter (Mindray BC*‐*6800, Mindray Bio‐Medical Electronics Co., Ltd., Shenzhen, China). Hb typing was performed using an automated HPLC instrument (VARIANT^TM^, Bio-Rad Laboratories, Hercules, CA). α-Thalassemia genotype was performed to detect both deletion and non-deletion α-thalassemias. The deletion type, α^0^-thalassemia (--^SEA^,--^THAI^) and α^+^-thalassemia (-α^3.7^, -α^4.2^) were carried out by GAP-PCR [30]. The non-deletion type, Hb Constant Spring and Hb Pakse were genotyped by dot-blot hybridization [31].

### Human ethics

Ethical approval for this study was obtained from the Ethics Committee, Faculty of Associated Medical Sciences, Chiang Mai University (AMSEC-60EX-022). The samples in this study were the routinely leftover blood samples and were used anonymously to maintain confidentiality.

## Results

### Identification of an appropriate monoclonal antibody pair for the establishment of an immunochromatographic strip test

Two anti-ζ-globin chain mAb clones, named PL2 and PL3, were established in our research center [28, 32] and used to develop an IC strip test for the detection of ζ-globin chains.

**Fig 1:**
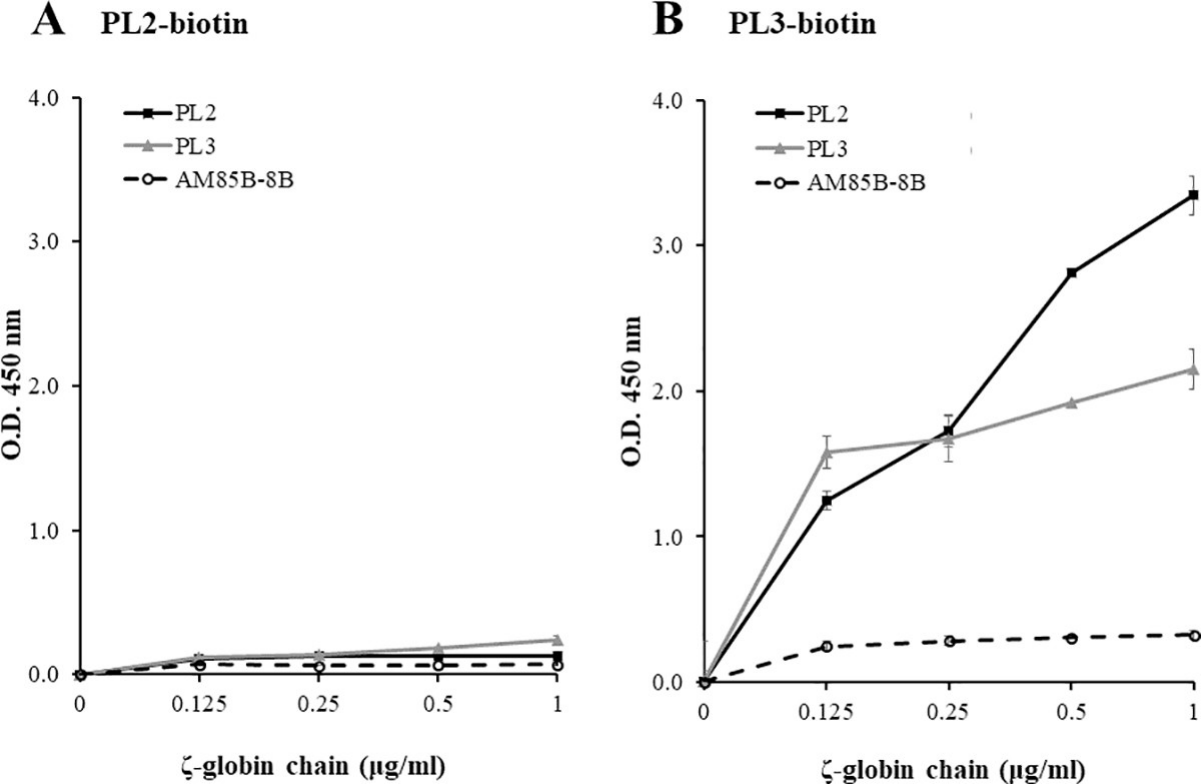
Identification of an anti-ζ-globin chain mAbs pair for detecting ζ-globin chain. **An** ELISA plate was coated with 10 μg/mL of the anti-ζ-globin chain mAb clones PL2 or PL3 or an isotype-matched control mAb (AM85B-8B) as indicated. A sandwich ELISA for detecting various concentrations of ζ-globin chains was performed using (A) biotin-labeled mAb PL2 (PL2-biotin) and (B) biotin-labeled mAb PL3 (PL3-biotin). HRP-conjugated streptavidin was used to monitor the antigen-antibody reaction.

Prior to the IC strip test development, experiments were performed to identify the proper anti-ζ-globin chain mAbs to use as detecting or capturing mAbs. Anti-ζ-globin chain mAb clones PL2 and PL3 and an isotype-matched control mAb (AM85B-8B) were coated on an ELISA plate as capturing mAbs. Various concentrations of ζ-globin chains were added into the ELISA plates. Biotin-labeled mAb PL2 (PL2-biotin) or PL3 (PL3-biotin) were added as detecting mAbs to detect the bound ζ-globin chains. Using the mAb PL2-biotin as a detecting mAb, ζ-globin chains could not be detected when using either mAb PL2 or PL3 as a capturing mAb (Fig 1A). In contrast, the mAb PL3-biotin showed positive reactivity with either capturing mAb PL2 or PL3 in a dose-dependent manner (Fig 1B). The results indicated that mAb PL2 as a capturing mAb and mAb PL3 as detecting mAb was the appropriate mAb pair for detecting ζ-globin chains in a sandwich type immunoassay format.

### Construction of an immunochromatographic strip test for detecting ζ-globin chains

**Fig 2:**
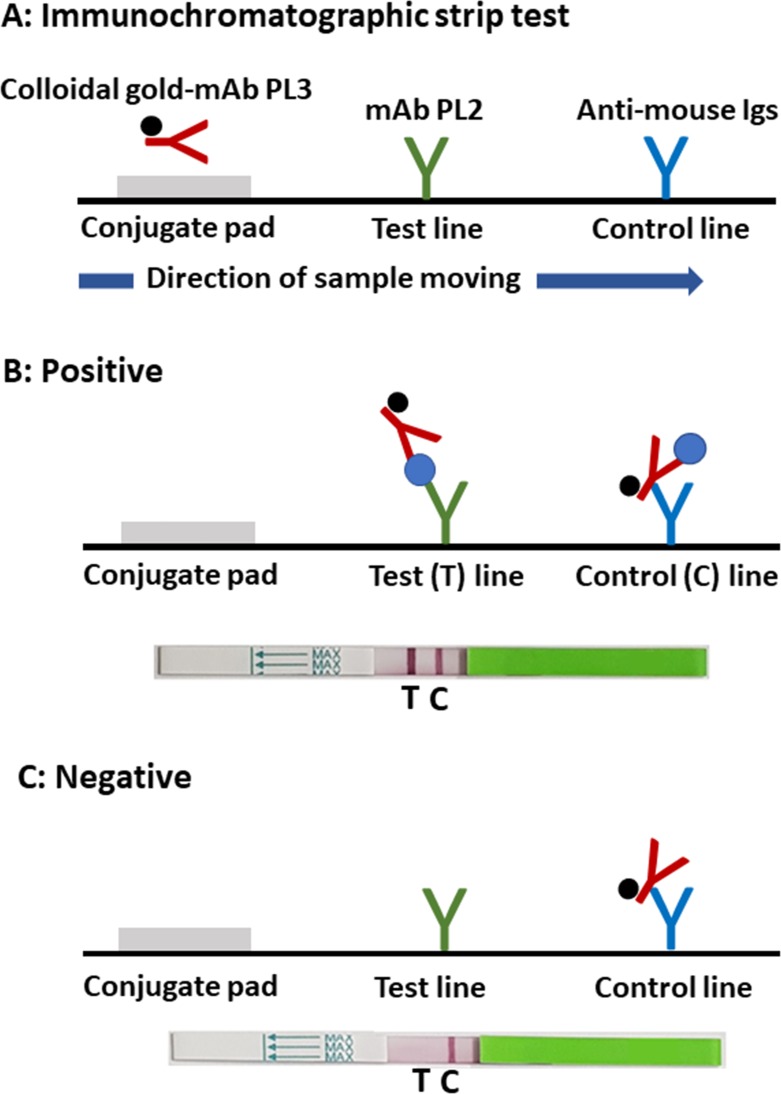
Schematic diagram demonstrating the principle of the immunochromatographic strip test for the detection of ζ-globin chains. (A) The IC test strip consists of four components: a sample application pad, a conjugate pad, an analytical nitrocellulose membrane and an absorbent pad. Colloidal gold-conjugated anti-ζ-globin mAb clone PL3 was absorbed at the conjugate pad. The anti-ζ-globin chain mAb clone PL2 and anti-mouse immunoglobulins antibody were immobilized on the nitrocellulose membrane at the test (T) and control (C) line zones, respectively. Subsequently, the components of the strip test were assembled and then cut into individual strips. (B) A strip showing the red-purple streak at the test (T) and control (C) line zones is interpreted as a positive result. (C) A negative result appears as only a red-purple streak at the control (C) line zone.

According to the results obtained above, in the IC strip test development, the mAbs PL2 and PL3 were used as capturing and detecting mAbs, respectively. The schematic representation of the developed IC strip test for the detection of ζ-globin chains in blood samples (named the IC ζ strip test) is shown in Fig 2A. Conjugation of colloidal gold with the anti-ζ-globin chain mAb clone PL3 was produced as described elsewhere [21] and absorbed and dried at the conjugate pad. The anti-ζ-globin chain mAb clone PL2 and anti-mouse immunoglobulin antibodies were immobilized at the test (T) and control (C) line zones, respectively. For determining the presence of ζ-globin chains, the IC strip was vertically immersed in hemolyzed blood [21] for 5 minutes. The reactive bands on the strip were visualized by eye at the T and C line zones. For positive reactivity, 2 red-purple bands were detected at both the T and C line zones (Fig 2B). For negative reactivity, only 1 red-purple band was detected at the C line zone (Fig 2C).

**Fig 3:**
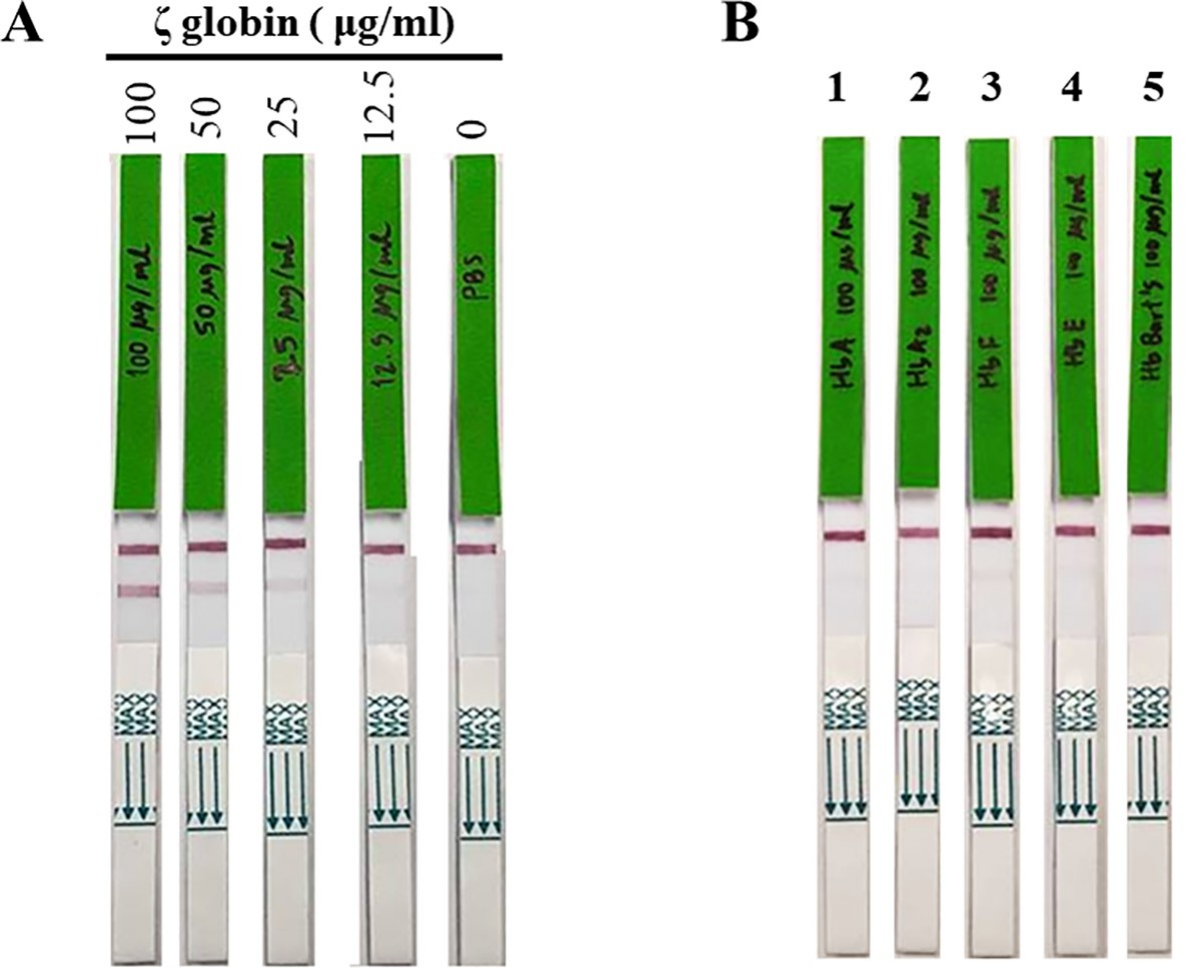
Sensitivity and specificity of the immunochromatographic strip test for the detection of ζ-globin chains. (A) Various concentrations of ζ-globin chains and (B) Hb Bart’s, Hb A, Hb A_2_, Hb F and Hb E at 100 μg/mL were assayed using the established IC ζ strips. The reactive bands on the strip were observed by naked eye at the test and control line zones.

To determine the analytical sensitivity and specificity of the generated IC ζ strip test, hemolysates containing various concentrations of ζ-globin chains and 100 μg/mL of purified Hb Bart’s, Hb A, Hb A_2_, Hb F and Hb E were tested. The results of analytical sensitivity and analytical specificity analyses of the IC ζ strips are shown in Fig 3. The sensitivity of the IC test strip for detecting ζ-globin chains was 25 μg/mL (Fig 3A). Purified Hb Bart’s, Hb A, Hb A_2_, Hb F and Hb E (at 100 μg/mL) did not generate positive reactivity (Fig 3B).

### Validation of the immunochromatographic strip tests for screening α^0^-thalassemia carriers

In this study, the IC ζ strip test for detecting ζ-globin chains was validated in parallel with the commercialized IC strip test for detecting Hb Bart’s (i+LAB α THAL IC strip test [21, 25–27].

Blood samples of various thalassemia and non-thalassemia subjects (n = 195) were recruited for this validation (Table 1). The IC ζ and i+LAB α THAL IC strip test results for each sample are also shown in Table 1. The hematologic parameters (MCV/MCH) could be used for the differentiation of thalassemia and normal subjects. MCV ≥ 80 fL and MCH ≥ 27 pg were the cutoff values [33, 34]. Among the 195 recruited samples in this study, 90 samples were MCV ≥ 80 fL and MCH ≥ 27 pg (high MCV/MCH), while 105 samples were MCV < 80 fL or MCH < 27 pg (low MCV/MCH) (Table 1). All α^0^-thalassemia traits exhibited low MCV or MCH values. Among the 105 samples with low MCV/MCH values, 78 samples carried the α^0^-thalassemia (--^SEA^) gene and 4 samples carried the α^0^-thalassemia (--^THAI^) gene (Table 1).

**Table 1:** The hematologic parameters (MCV and MCH) and IC strip test results for 195 blood samples.

**MCV ≥ 80 fL and MCH ≥ 27 pg (High MCV/MCH)**					
**Genotype**	**No.**	**IC strip**			
		**ζ-globin**		**Hb Bart’s**	
		**+**	**-**	**+**	**-**
Normal hemoglobin type α^+^-thalassemia heterozygote HbE heterozygote HbE heterozygote with α^+^-thalassemia heterozygote Hb CS Homozygous Hb CS heterozygote HbE heterozygote with Hb CS heterozygote α^+^-thalassemia heterozygote with Hb CS heterozygote	48 17 10 3 1 9 1 1	5 7 2 1 0 1 0 1	43 10 8 2 1 8 1 0	1 8 2 0 1 9 1 1	47 9 8 3 0 0 0 0
Total	90	17	73	23	67
**MCV < 80 fL or MCH < 27 pg (Low MCV/MCH)**					
**Genotype**	**No.**	**IC strip**			
		**ζ-globin**		**Hb Bart’s**	
		**+**	**-**	**+**	**-**
β-thalassemia/HbE disease β-thalassemia heterozygote β-thalassemia heterozygote with α^+^-thalassemia heterozygote HbE homozygous HbE homozygous with α^+^-thalassemia heterozygote HbE heterozygote with α^+^-thalassemia homozygote β-thalassemia homozygote with α^+^-thalassemia heterozygote α^+^-thalassemia homozygote α^0^-thalassemia heterozygote HbE heterozygote with α^0^-thalassemia heterozygote HbH disease HbH-CS disease EA Bart’s disease (Hb H disease with Hb E trait) CSEA Bart’s disease (Hb H-CS with Hb E trait) β-thalassemia/HbE disease with α^0^-thalassemia heterozygote HbH disease (α^0^-thalassemia (--^THAI^) HbH-CS disease (α^0^-thalassemia (--^THAI^))	1 4 2 5 2 2 1 6 36 5 13 15 2 5 2 3 1	0 1 1 1 2 0 0 3 36 5 13 15 2 5 2 0 0	1 3 1 4 0 2 1 3 0 0 0 0 0 0 0 3 1	0 0 1 1 1 2 1 6 36 4 13 15 1 5 1 3 1	1 4 1 4 1 0 0 0 0 1 0 0 1 0 1 0 0
Total	105	86	19	91	14

One hundred ninety-five subjects with various thalassemia and normal hemoglobin were recruited in this study. Ninety samples were MCV ≥ 80 fL and MCH ≥ 27 pg (High MCV/MCH); 105 samples were MCV < 80 fL or MCH < 27 pg (Low MCV/MCH), as indicated. The IC strip test results (+, positive; -, negative) of each subject are indicated.

All subjects with the α^0^-thalassemia gene are SEA deletion type (--^SEA^), except those that are indicated as α^0^-thalassemia (--^THAI^).

For screening α^0^-thalassemia carriers using the established IC strip tests, MCV/ MCH values were used to exclude non-α^0^-thalassemia subjects, and 90 out of 195 samples were ruled out. Among the remaining 105 samples having low MCV or MCH values, 4 samples containing the α^0^-thalassemia (--^THAI^) gene, which is a non-SEA deletion type, were omitted. Therefore, the remaining 101 samples were used to analyze the clinical sensitivity, clinical specificity, PPV and NPV of the IC strip tests for the identification of the α^0^-thalassemia (--^SEA^) trait. Using IC ζ strip tests, the sensitivity, specificity, PPV and NPV for the screening of α^0^-thalassemia (--^SEA^) traits were measured as 100, 65.2, 90.7 and 100%, respectively (Table 2). Using the i+LAB α THAL IC strip test, the sensitivity, specificity, PPV and NPV for the screening of α^0^-thalassemia (--^SEA^) traits were measured as 96.2, 47.8, 86.2 and 78.6%, respectively (Table 3).

**Table 2:** Clinical sensitivity, clinical specificity, positive predictive value (PPV) and negative predictive value (NPV) of immunochromatographic strip tests for ζ-globin chains (IC ζ strip test). All samples in the Table have low MCV/MCH values (MCV < 80 fL or MCH < 27 pg). The α^0^-thalassemia (--^THAI^) subjects were excluded from this analysis.

IC ζ strip test	α^0^-thalassemia (SEA deletion type) assayed by PCR		
	Positive	Negative	Total
Positive	78	8	86
Negative	0	15	15
Total	78	23	101

Sensitivity of IC ζ strip test = (78/78) × 100 = 100%

Specificity of IC ζ strip test = (15/23) × 100 = 65.2%

Positive predictive value of IC ζ strip test = (78/86) × 100 = 90.7%

Negative predictive value of IC ζ strip test = (15/15) × 100 = 100%

**Table 3:** Clinical sensitivity, clinical specificity, positive predictive value (PPV) and negative predictive value (NPV) of immunochromatographic strip tests for Hb Bart’s (i+LAB α THAL IC strip test). All samples in the Table have low MCV/MCH values (MCV < 80 fL or MCH < 27 pg). The α^0^-thalassemia (--^THAI^) subjects were excluded from this analysis.

i+LAB α THAL IC strip test	α^0^-thalassemia (SEA deletion type) assayed by PCR		
	Positive	Negative	Total
Positive	75	12	87
Negative	3	11	14
Total	78	23	101

Sensitivity of i+LAB α THAL IC strip test = (75/78) × 100 = 96.2%

Specificity of i+LAB α THAL IC strip test = (11/23) × 100 = 47.8%

Positive predictive value of i+LAB α THAL IC strip test = (75/87) × 100 = 86.2%

Negative predictive value of i+LAB α THAL IC strip test = (11/14) × 100 = 78.6%

If all subjects were analyzed, excluding α^0^-thalassemia (--^THAI^) (191 samples), the sensitivity, specificity, PPV and NPV of the IC ζ and i+LAB α THAL IC strip tests for the screening of α^0^-thalassemia (--^SEA^) traits were 100, 77.8, 75.7, and 100% and as 96.2, 69.0, 68.2, and 96.3%, respectively (Tables 4 and 5).

**Table 4:** Clinical sensitivity, clinical specificity, positive predictive value (PPV) and negative predictive value (NPV) of immunochromatographic strip tests for ζ-globin chains (IC ζ strip test). All subjects recruited in this study are shown. The 4 α^0^-thalassemia (--^THAI^) subjects were excluded from this analysis.

IC ζ strip test	α^0^-thalassemia (SEA deletion type) assayed by PCR		
	Positive	Negative	Total
Positive	78	25	103
Negative	0	88	88
Total	78	113	191

Sensitivity of IC zeta strip test = (78/78) × 100 = 100%

Specificity of IC zeta strip test = (88/113) × 100 = 77.8%

Positive predictive value of IC zeta strip test = (78/103) × 100 = 75.7%

Negative predictive value of IC zeta strip test = (88/88) × 100 = 100%

**Table 5:** Clinical sensitivity, clinical specificity, positive predictive value (PPV) and negative predictive value (NPV) of immunochromatographic strip tests for Hb Bart’s (i+LAB α THAL IC strip test). All subjects recruited in this study are shown. The 4 α^0^-thalassemia (--^THAI^) subjects were excluded from this analysis.

i+LAB α THAL IC strip test	α^0^-thalassemia (SEA deletion type) assayed by PCR		
	Positive	Negative	Total
Positive	75	35	110
Negative	3	78	81
Total	78	113	191

Sensitivity of i+LAB α THAL IC strip test = (75/78) × 100 = 96.2%

Specificity of i+LAB α THAL IC strip test = (78/113) × 100 = 69.0%

Positive predictive value of i+LAB α THAL IC strip test = (75/110) × 100 = 68.2%

Negative predictive value of i+LAB α THAL IC strip test = (78/81) × 100 = 96.3%

As expected, all α^0^-thalassemia (--^THAI^) (n = 4) tested positive using the i+LAB α THAL IC strip test but tested negative using the IC ζ strip tests (Table 1). Thus, the α^0^-thalassemia (--^THAI^) and α^0^-thalassemia (--^SEA^) carriers could be discriminated using these two IC strip tests.

### α^0^-Thalassemia screening strategy using immunochromatographic strip tests for ζ-globin chains and Hb Bart’s

As hematologic parameters and Hb typing are unable to differentiate α^0^-thalassemia, α^+^-thalassemia and normal subjects, genotyping via PCR is currently required for the diagnosis of α-thalassemia. Based on the results obtained in the previous section and to reduce the cost of genotyping, we proposed to include IC ζ and i+LAB α THAL IC strip tests within a α^0^-thalassemia screening strategy as follows.

**Fig 4:**
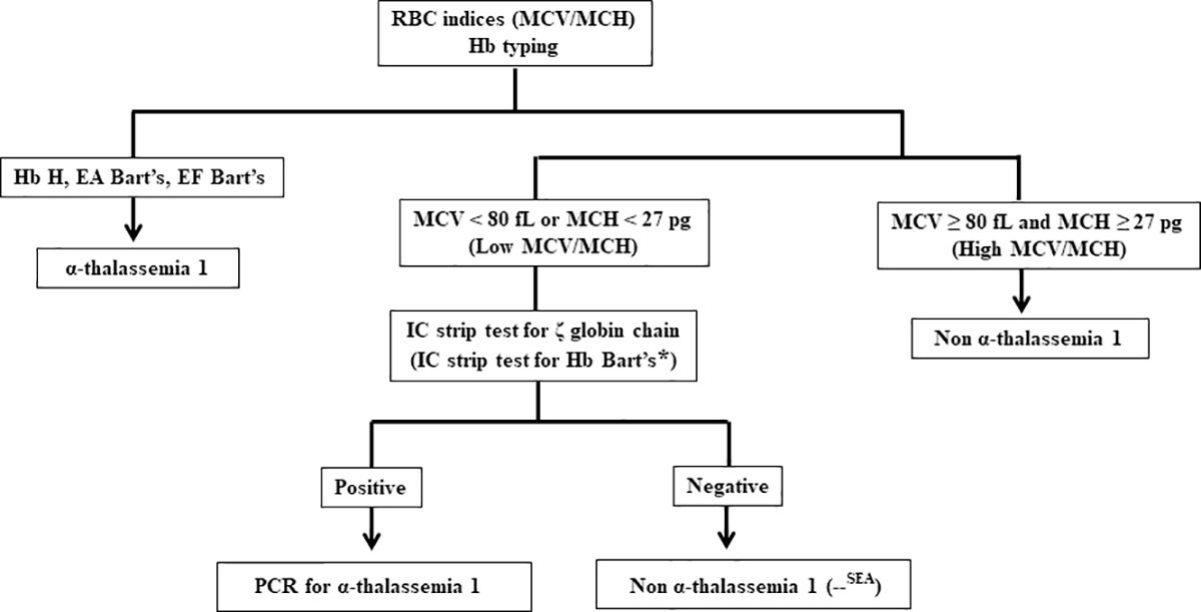
A proposed α^0^-thalassemia screening strategy. The hematologic analysis, including MCV/MCH and Hb typing, are first performed for each blood sample. Notably, the Hb typing is not necessary in screening of α^0^-thalassemia traits only. Samples with high MCV and MCH values are ruled out as α^0^-thalassemia carriers. The IC strip test for ζ-globin chains (and IC strip test for Hb Bart’s) is subsequently performed using samples with low MCV or MCH values. The IC strip test negative samples can be ruled out for α^0^-thalassemia (--^SEA^). The positive samples are then subjected to α^0^-thalassemia genotyping by PCR. The samples identified as Hb H, EA Bart’s (Hb H disease with Hb E trait) and EF Bart’s diseases (Hb H disease with β^0^-thalassemia/Hb E or Hb H disease with homozygous Hb E) do not need to be assayed using the IC strip test assay. Samples with a non-SEA deletion type (α^0^-thalassemia (--^THAI^)) will test negative using the IC strip test for ζ-globin chains. To identify this type of α^0^-thalassemia, an IC strip test for Hb Bart’s is required. * To determine or differentiate α^0^-thalassemia (--^THAI^) and α^0^-thalassemia (--^SEA^), an IC strip test for Hb Bart’s is required.

In Southeast Asian countries, α^0^-thalassemia (--^SEA^) represents the majority of α^0^-thalassemia traits [2, 8, 12, 19, 20]. For this region, we propose an α^0^-thalassemia screening strategy as shown in Fig 4. The hematologic analysis, including MCV/MCH and Hb typing, are first performed for each blood sample. It is noted that the Hb typing is used for determination of β-thalassemia. Samples with high MCV/MCH values (MCV ≥ 80 fL and MCH ≥ 27 pg) are indicated as non-α^0^-thalassemia carriers. The IC ζ strip tests are subsequently performed using only those samples having low MCV/MCH values (MCV < 80 fL or MCH < 27 pg). As the sensitivity and NPV of this strip test were 100%, the IC strip test negative samples can be ruled out for having the α^0^-thalassemia (--^SEA^) gene. As the specificity of the IC strip test was approximately 65%, the positive samples are then subjected to α^0^-thalassemia genotyping by PCR. Additionally, the samples identified as Hb H, Hb H-CS, EA Bart’s or CSEA Bart’s diseases by Hb typing are not necessary for IC strip test assay as they would carry the α^0^-thalassemia gene. Notably, samples having α^0^-thalassemia (--^THAI^), which is non-SEA deletion type, test negative using the IC ζ strip test (Table 1). To identify this type of α^0^-thalassemia, the i+LAB α THAL IC strip test is necessary and can differentiate between α^0^-thalassemia (--^THAI^) and α^0^-thalassemia (--^SEA^).

## Discussion

In this study, using our generated anti-ζ-globin chain mAbs [28], an IC strip test for ζ-globin chains (IC ζ strip test) was established. The IC ζ and commercial i+LAB α THAL IC strip tests were validated simultaneously using samples from various thalassemia and non-thalassemia subjects. All α^0^-thalassemia (--^SEA^) samples tested positive using the IC ζ strip test. These results were in accordance with previous reports showing that small amounts of embryonic ζ-globin chains are present in hemolysates and can serve as a marker for (--^SEA^) α^0^-thalassemia traits [32, 35, 36]. Accordingly, the negative IC ζ strip test results could exclude α^0^-thalassemia (--^SEA^) carriers. However, using the i+LAB α THAL IC strip test, 3 α^0^-thalassemia (--^SEA^) samples showed a negative result. As Hb Bart’s has also been demonstrated as a marker for α^0^-thalassemia traits [21, 25–27], the negative samples in this study may be false negatives. It is worth noting that these 3 samples showed a very faint band in the T line zone but were designed as negative. Thus, in our study, the i+LAB α THAL IC strip test had a slightly lower clinical sensitivity than the IC ζ strip test (96% vs. 100%) in the identification of α^0^-thalassemia traits (--^SEA^).

The SEA type deletion (--^SEA^) is the most common type of α^0^-thalassemia in Southeast Asian countries and southern China [2, 8, 11, 17, 18]. However, a very rare non-SEA type α^0^-thalassemia exists in which the ζ -globin gene is deleted, [24]. This type of α^0^-thalassemia, including α^0^-thalassemia (--^THAI^), does not result in ζ-globin chain production. Interestingly, the 4 α^0^-thalassemia (--^THAI^) subjects recruited in this study tested negative using the IC ζ strip tests and positive with the i+LAB α THAL IC strip test. Thus, the i+LAB α THAL IC strip test was capable of identifying these rare α^0^-thalassemia defects. Among subjects carrying a non-α^0^-thalassemia gene (including α^+^-thalassemia, β-thalassemia and Hb E) and a normal hemoglobin type, the IC ζ and i+LAB α THAL IC strip tests showed irregular patterns that were independent to their α- or β-globin gene abnormalities. However, the majority of normal subjects tested negative using both IC strip tests. These results are similar to those obtained previously [21, 25–27, 35, 36]. The cause of the positivity of the IC strip tests with these subjects is still unknown and may be due to cross-reactivity of mAbs used in the IC strip tests to other Hbs [35]. Although the IC strip tests could detect other thalassemias in addition to α^0^-thalassemia, this test will have a great benefit for the identification of β-thalassemia subjects that also have α-thalassemia genes [26].

Identification of α^0^-thalassemia carriers is an essential part of preventing severe α-thalassemia disease, and DNA-based analysis of gene deletions by PCR is currently the most accurate diagnosis method [13–16]. However, PCR has specific significant limitations, including high cost and the need for sophisticated laboratory instrumentation and well-trained technicians. These factors prevent PCR from being widely used for α^0^-thalassemia screening, especially in rural areas or in resource-limited countries. According to the validation results of our established IC strip tests, the α^0^-thalassemia screening strategy was proposed for Southeast Asian countries, where α^0^-thalassemia (--^SEA^) predominates (Fig 4). MCV ≥ 80 fL and MCH ≥ 27 pg were suggested to be the cut-off values for discriminating normal or non-clinically significant thalassemia from thalassemia subjects [33, 34]. In our study, MCV ≥ 80 fL and MCH ≥ 27 pg cut-off values also ruled out 100% of α^0^-thalassemia. Therefore, the MCV/MCH analyses were included in our proposed screening strategy, where blood samples will be first determined by hematologic analysis including MCV/MCH. Blood samples with both high MCV and MCH values are excluded for the presence of α^0^-thalassemia gene and do not need to be assessed by an IC strip assay. Samples with low MCV or MCH values are then assayed using the IC ζ strip test (or both the IC ζ and i+LAB α THAL IC strip tests). The positive samples for the IC strip test are recommended for further PCR analysis for α^0^-thalassemia. Using this strategy, the cost for PCR for large-scale α^0^-thalassemia screening analyses will be reduced. It is worth noting that the IC ζ strip test does not detect the non-SEA deletion type of α^0^-thalassemia, such as the (--^THAI^) and (--^FIL^) types. However, in the Southeast Asian region, the prevalence of these gene deletions is very low [37]. For α^0^-thalassemia (--^THAI^) and (--^FIL^), the PCR analysis is recommended for α^0^-thalassemia characterization. The low MCV and MCH values can certainly be observed in iron deficiency anemia. However, the effect of the reduced iron store on the performance of the established IC ζ strip test was not yet verified. Nevertheless, MCV and MCH values can be increased under many conditions, potentially affecting the validity of the proposed α^0^-thalassemia screening strategy.

In our setting, the cost of PCR for α-thalassemia DNA analysis is USD $15, whereas the cost of the IC ζ strip test is approximately USD $5. Accordingly, the cost-effectiveness of the proposed α^0^-thalassemia screening strategy was determined. For example, in this study, 195 blood samples were studied. If all subjects were routinely screened for α^0^-thalassemia (--^SEA^) by PCR, this would cost USD $2,925 (195 tests × USD $15). However, using our strategy, 90 out of 195 samples that were MCV ≥ 80 fL and MCH ≥ 27 pg could be excluded for α^0^-thalassemia subjects. The remaining samples (105 samples) were screened using the IC ζ strip test, which would cost USD $525 (105 tests × USD $5). Among the 105 samples, 78 tested positive using the IC ζ strip test. Therefore, these samples, therefore did not require further PCR screening. The remaining 27 samples were then confirmed by PCR, costing USD $405 (27 tests × US$15). Therefore, using our strategy, 168 samples (out of 198) could be excluded for analysis using the expensive and sophisticated PCR method. Accordingly, of these 198 samples, approximately USD $1,000 would be saved when using the proposed α^0^-thalassemia screening strategy compared to the conventional PCR-based protocol.

## Conclusions

In summary, we have developed two types of IC strip tests, one for the detection of Hb Bart’s and another for ζ-globin chains. The IC strip tests are very easy to perform, and the results can be visually interpreted without an expert. Therefore, the IC strip test is suitable for use in testing a large number of samples. The established IC strip tests are suggested to be included in the α-thalassemia screening strategy. In combination with the results of hematological analysis, the IC strip tests can rule out a mass population for further α^0^-thalassemia detection by PCR-based analysis. Using our proposed α-thalassemia screening strategy, the cost for the diagnosis of α^0^-thalassemia carriers will be reduced and is appropriate for Southeast Asian countries. The developed IC strip tests for Hb Bart’s and ζ-globin chains are point-of-care testing (POCT) methods that are applicable for every hospital level.

## Supporting information

S1 Table The genotypes, phenotypes, IC strip test results and hematologic parameters (MCV and MCH) of the 195 blood samples. (DOCX)

## References

[1] Weatherall DJ, Clegg JB. The Thalassemia Syndromes. 4th ed: Wiley-Blackwell; 2001.

[2] Harteveld CL, Oosterhuis WP, Schoenmakers CH, Ananta H, Kos S, Bakker Verweij M, et al alpha-thalassaemia masked by beta gene defects and a new polyadenylation site mutation on the alpha2-globin gene. Eur J Haematol. 2010;84(4):354–8. 10.1111/j.1600-0609.2009.01380.x . 19912309

[3] Galanello R, Cao A. Gene test review. Alpha-thalassemia. Genetics in medicine: official journal of the American College of Medical Genetics. 2011;13(2):83–8. 10.1097/GIM.0b013e3181fcb468 . 21381239

[4] Leung WC, Leung KY, Lau ET, Tang MH, Chan V. Alpha-thalassaemia. Semin Fetal Neonatal Med. 2008;13(4):215–22. 10.1016/j.siny.2008.02.006 . 18406222

[5] Chui DH. Alpha-thalassaemia and population health in Southeast Asia. Ann Hum Biol. 2005;32(2):123–30. 10.1080/03014460500075084 . 16096207

[6] Chui DH. Alpha-thalassemia: Hb H disease and Hb Barts hydrops fetalis. Annals of the New York Academy of Sciences. 2005;1054:25–32. 10.1196/annals.1345.004 . 16339648

[7] Liao C, Pan M, Han J, Yang X, Zhen L, Li J, et al Prenatal control of Hb Bart's hydrops fetalis: a two-year experience at a mainland Chinese hospital. The journal of maternal-fetal & neonatal medicine: the official journal of the European Association of Perinatal Medicine, the Federation of Asia and Oceania Perinatal Societies, the International Society of Perinatal Obstet. 2015;28(4):413–5. 10.3109/14767058.2014.918597 . 24766075

[8] Fucharoen S, Winichagoon P, Thonglairoam V, Siriboon W, Siritanaratkul N, Kanokpongsakdi S, et al Prenatal diagnosis of thalassemia and hemoglobinopathies in Thailand: experience from 100 pregnancies. Southeast Asian J Trop Med Public Health. 1991;22(1):16–29. . 1948258

[9] Weatherall DJ. The inherited diseases of hemoglobin are an emerging global health burden. Blood. 2010;115(22):4331–6. 10.1182/blood-2010-01-251348 20233970 PMC2881491

[10] Piel FB, Weatherall DJ. The alpha-thalassemias. N Engl J Med. 2014;371(20):1908–16. 10.1056/NEJMra1404415 . 25390741

[11] Chui DH, Waye JS. Hydrops fetalis caused by alpha-thalassemia: an emerging health care problem. Blood. 1998;91(7):2213–22. . 9516118

[12] Weatherall D. The inherited disorders of haemoglobin: an increasingly neglected global health burden. Indian J Med Res. 2011;134:493–7. 22089613 PMC3237249

[13] Chang JG, Lee LS, Lin CP, Chen PH, Chen CP. Rapid diagnosis of alpha-thalassemia-1 of southeast Asia type and hydrops fetalis by polymerase chain reaction. Blood. 1991;78(3):853–4. . 1859898

[14] Chong SS, Boehm CD, Higgs DR, Cutting GR. Single-tube multiplex-PCR screen for common deletional determinants of alpha-thalassemia. Blood. 2000;95(1):360–2. . 10607725

[15] Hartwell SK, Srisawang B, Kongtawelert P, Christian GD, Grudpan K. Review on screening and analysis techniques for hemoglobin variants and thalassemia. Talanta. 2005;65(5):1149–61. 10.1016/j.talanta.2004.09.013 . 18969925

[16] Panyasai S, Sringam P, Fucharoen G, Sanchaisuriya K, Fucharoen S. A simplified screening for alpha-thalassemia 1 (SEA type) using a combination of a modified osmotic fragility test and a direct PCR on whole blood cell lysates. Acta Haematol. 2002;108(2):74–8. 10.1159/000064746 . 12187024

[17] Cohen AR, Galanello R, Pennell DJ, Cunningham MJ, Vichinsky E. Thalassemia. Hematology Am Soc Hematol Educ Program. 2004:14–34. 10.1182/asheducation-2004.1.14 . 15561674

[18] Cao A, Kan YW. The prevention of thalassemia. Cold Spring Harb Perspect Med. 2013;3(2):a011775 10.1101/cshperspect.a011775 23378598 PMC3552345

[19] Wasi P, Pravatmuang P, Winichagoon P. Immunologic diagnosis of alpha-thalassemia traits. Hemoglobin. 1979;3(1):21–31. 10.3109/03630267909069152 . 457421

[20] Makonkawkeyoon L, Ongchai S, Sanguansermsri T. Determination of hemoglobin Bart's in alpha thalassemia traits by two-site immunoradiometric assay. II. Detection of hemoglobin Bart's in alpha thalassemia traits. J Med Assoc Thai. 1992;75(10):565–9. . 1306192

[21] Tayapiwatana C, Kuntaruk S, Tatu T, Chiampanichayakul S, Munkongdee T, Winichagoon P, et al Simple method for screening of alpha-thalassaemia 1 carriers. Int J Hematol. 2009;89(5):559–67. 10.1007/s12185-009-0331-4 . 19440681

[22] Kutlar F, Moscoso H, Kiefer CR, Garver FA, Beksac S, Onderoglu L, et al Quantities of adult, fetal and embryonic globin chains in the blood of eighteen- to twenty-week-old human fetuses. J Chromatogr. 1991;567(2):359–68. 10.1016/0378-4347(91)80142-y . 1939469

[23] Chui DH, Wong SC, Chung SW, Patterson M, Bhargava S, Poon MC. Embryonic zeta-globin chains in adults: a marker for alpha-thalassemia-1 haplotype due to a greater than 17.5-kb deletion. N Engl J Med. 1986;314(2):76–9. 10.1056/NEJM198601093140203 . 3941693

[24] Tang W, Luo HY, Albitar M, Patterson M, Eng B, Waye JS, et al Human embryonic zeta-globin chain expression in deletional alpha-thalassemias. Blood. 1992;80(2):517–22. . 1627804

[25] Bunkall C, Ghallyan N, Elliott C, Van de Water N, Chan G. Evaluation of an immunochromatographic strip test for alpha-thalassaemia screening. Int J Lab Hematol. 2018;40(6):691–6. 10.1111/ijlh.12905 . 30118579

[26] Winichagoon P, Kumpan P, Holmes P, Finlayson J, Newbound C, Kabral A, et al Validation of the immunochromatographic strip for alpha-thalassemia screening: a multicenter study. Transl Res. 2015;165(6):689–95. 10.1016/j.trsl.2014.10.013 . 25450870

[27] Prayalaw P, Fucharoen G, Fucharoen S. Routine screening for alpha-thalassaemia using an immunochromatographic strip assay for haemoglobin Bart's. J Med Screen. 2014;21(3):120–5. 10.1177/0969141314538611 . 24907301

[28] Pata S, Khummuang S, Pornprasert S, Tatu T, Kasinrerk W. A simple and highly sensitive ELISA for screening of the alpha-thalassemia-1 Southeast Asian-type deletion. J Immunoassay Immunochem. 2014;35(2):194–206. 10.1080/15321819.2013.838963 . 24295182

[29] Phunpae P, Chanwong S, Tayapiwatana C, Apiratmateekul N, Makeudom A, Kasinrerk W. Rapid diagnosis of tuberculosis by identification of Antigen 85 in mycobacterial culture system. Diagn Microbiol Infect Dis. 2014;78(3):242–8. 10.1016/j.diagmicrobio.2013.11.028 . 24418370

[30] Chong SS, Boehm CD, Cutting GR, Higgs DR. Simplified multiplex-PCR diagnosis of common southeast asian deletional determinants of alpha-thalassemia. Clin Chem. 2000;46(10):1692–5. Epub 2000/10/06. . 11017952

[31] Pichanun D, Munkongdee T, Klamchuen S, Butthep P, Winichagoon P, Fucharoen S, et al Molecular screening of the Hbs Constant Spring (codon 142, TAA>CAA, alpha2) and Pakse (codon 142, TAA>TAT, alpha2) mutations in Thailand. Hemoglobin. 2010;34(6):582–6. Epub 2010/11/17. 10.3109/03630269.2010.526914 . 21077767

[32] Pata S, Pongpaiboon M, Laopajon W, Munkongdee T, Paiboonsukwong K, Pornpresert S, et al Immunostick Test for Detecting zeta-Globin Chains and Screening of the Southeast Asian alpha-Thalassemia 1 Deletion. Biol Proced Online. 2019;21:15 Epub 2019/08/08. 10.1186/s12575-019-0104-2 31388336 PMC6670165

[33] Sanchaisuriya K, Fucharoen S, Fucharoen G, Ratanasiri T, Sanchaisuriya P, Changtrakul Y, et al A reliable screening protocol for thalassemia and hemoglobinopathies in pregnancy: an alternative approach to electronic blood cell counting. Am J Clin Pathol. 2005;123(1):113–8. 10.1309/fuf9evgq24v1pktp . 15762286

[34] Guidelines for investigation of the alpha and beta thalassaemia traits. The Thalassaemia Working Party of the BCSH General Haematology Task Force. J Clin Pathol. 1994;47(4):289–95. 10.1136/jcp.47.4.289 7517954 PMC501928

[35] Jomoui W, Fucharoen G, Sanchaisuriya K, Fucharoen S. Screening of (-SEA) alpha-thalassaemia using an immunochromatographic strip assay for the zeta-globin chain in a population with a high prevalence and heterogeneity of haemoglobinopathies. J Clin Pathol. 2017;70(1):63–8. 10.1136/jclinpath-2016-203765 . 27312111

[36] Wen L, Zhu P, Liu Y, Pan Q, Qu Y, Xu X, et al Development of a fluorescence immunochromatographic assay for the detection of zeta globin in the blood of (--(SEA)) alpha-thalassemia carriers. Blood Cells Mol Dis. 2012;49(3–4):128–32. 10.1016/j.bcmd.2012.05.011 . 22677106

[37] Chaibunruang A, Prommetta S, Yamsri S, Fucharoen G, Sae-Ung N, Sanchaisuriya K, et al Molecular and hematological studies in a large cohort of alpha(0)-thalassemia in northeast Thailand: data from a single referral center. Blood Cells Mol Dis. 2013;51(2):89–93. 10.1016/j.bcmd.2013.04.003 . 23639268

