# Supplementary material for: Impact of the detection of ζ-globin chains and hemoglobin Bart’s using immunochromatographic strip tests for α0-thalassemia (--SEA) differential diagnosis
Source: PLoS One. 2019 Oct 29;14(10):e0223996. doi: 10.1371/journal.pone.0223996 (PMC6818768; doi:10.1371/journal.pone.0223996)
Supplement: S1 Table — (DOCX) [file pone.0223996.s001.docx]

**S1 Table:** **The genotypes, phenotypes, IC strip test results and hematologic parameters (MCV and MCH) of the 195 blood samples.**

| **Samples** | **Genotype** | **Phenotype*** | **IC stripe** | | **MCV/MCH**** | |
| --- | --- | --- | --- | --- | --- | --- |
|  |  |  | **ζ-globin** | **Hb Bart's** | **High** | **Low** |
| 1 | --^SEA^/α^CS^α | HbH-CS disease | Positive | Positive |  | 🗸 |
| 2 | --^SEA^/αα | Alpha-thalassemia 1 heterozygote | Positive | Positive |  | 🗸 |
| 3 | --^SEA^/α^CS^α | HbH-CS disease | Positive | Positive |  | 🗸 |
| 4 | --^SEA^/α^CS^α | HbH-CS disease | Positive | Positive |  | 🗸 |
| 5 | --^SEA^/α^CS^α | HbH-CS disease | Positive | Positive |  | 🗸 |
| 6 | --^SEA^/-α^3.7^ | HbH disease | Positive | Positive |  | 🗸 |
| 7 | --^THAI^/-α^3.7^ | HbH disease | Negative | Positive |  | 🗸 |
| 8 | --^THAI^/-α^3.7^ | HbH disease | Negative | Positive |  | 🗸 |
| 9 | --^SEA^/-α^3.7^ | HbH disease | Positive | Positive |  | 🗸 |
| 10 | --^SEA^/αα | Alpha-thalassemia 1 heterozygote | Positive | Positive |  | 🗸 |
| 11 | β^17^/β^N^, -α^3.7^/αα | Beta-thalassemia heterozygote with alpha-thalassemia 2 trait | Positive | Positive |  | 🗸 |
| 12 | β^17^/β^N^, αα/αα | Beta-thalassemia heterozygote | Negative | Negative |  | 🗸 |
| 13 | --^SEA^/α^CS^α | HbH-CS disease | Positive | Positive |  | 🗸 |
| 14 | β^E^/β^N^, --^SEA^/α^CS^α | CSEABart's disease | Positive | Positive |  | 🗸 |
| 15 | β^E^/β^E^, -α^3.7^/αα | Homozygous HbE with alpha-thalassemia 2 trait | Positive | Positive |  | 🗸 |
| 16 | β^17^/β^N^, αα/αα | Beta-thalassemia heterozygote | Negative | Negative |  | 🗸 |
| 17 | --^THAI^/-α^3.7^ | HbH disease | Negative | Positive |  | 🗸 |
| 18 | β^E^/β^N^, αα/αα | HbE heterozygote | Negative | Negative | 🗸 |  |
| 19 | α^CS^α/αα | Hb Constant Spring heterozygote | Negative | Positive | 🗸 |  |
| 20 | β^E^/β^N^, αα/αα | HbE heterozygote | Negative | Negative | 🗸 |  |
| 21 | β^4bp^/β^N^, αα/αα | Beta-thalassemia heterozygote | Positive | Negative |  | 🗸 |
| 22 | β^4bp^/β^E^, αα/αα | Beta-thalassemia/HbE disease | Negative | Negative |  | 🗸 |
| 23 | --^SEA^/α^CS^α | HbH-CS disease | Positive | Positive |  | 🗸 |
| 24 | α^CS^α/αα | Hb Constant Spring heterozygote | Negative | Positive | 🗸 |  |
| 25 | β^4bp^/β^4bp^, αα^74^/αα | Beta-thalassemia homozygote with alpha-thalassemia 2 trait | Negative | Positive |  | 🗸 |
| 26 | --^SEA^/α^CS^α | HbH-CS disease | Positive | Positive |  | 🗸 |
| 27 | --^SEA^/αα | Alpha-thalassemia 1 heterozygote | Positive | Positive |  | 🗸 |
| 28 | -α^3.7^/αα | Alpha-thalassemia 2 heterozygote | Negative | Positive | 🗸 |  |
| 29 | β^E^/β^E^, -α^3.7^/αα | Homozygous HbE with alpha-thalassemia 2 trait | Positive | Negative |  | 🗸 |
| 30 | --^SEA^/-α^3.7^ | HbH disease | Positive | Positive |  | 🗸 |
| 31 | --^SEA^/α^CS^α | HbH-CS disease | Positive | Positive |  | 🗸 |
| 32 | --^THAI^/α^CS^α | HbH-CS disease | Negative | Positive |  | 🗸 |
| 33 | --^SEA^/α^CS^α | HbH-CS disease | Positive | Positive |  | 🗸 |
| 34 | --^SEA^/αα | Alpha-thalassemia 1 heterozygote | Positive | Positive |  | 🗸 |
| 35 | -α^3.7^/αα | Alpha-thalassemia 2 heterozygote | Positive | Negative | 🗸 |  |
| 36 | --^SEA^/-α^3.7^ | HbH disease | Positive | Positive |  | 🗸 |
| 37 | --^SEA^/-α^4.2^ | HbH disease | Positive | Positive |  | 🗸 |
| 38 | --^SEA^/αα | Alpha-thalassemia 1 heterozygote | Positive | Positive |  | 🗸 |
| 39 | --^SEA^/-α^3.7^ | HbH disease | Positive | Positive |  | 🗸 |
| 40 | --^SEA^/α^CS^α | HbH-CS disease | Positive | Positive |  | 🗸 |
| 41 | --^SEA^/α^CS^α | HbH-CS disease | Positive | Positive |  | 🗸 |
| 42 | --^SEA^/α^CS^α | HbH-CS disease | Positive | Positive |  | 🗸 |
| 43 | β^E^/β^N^, --^SEA^/αα | HbE heterozygote with alpha-thalassemia 1 trait | Positive | Positive |  | 🗸 |
| 44 | β^E^/β^N^, --^SEA^/α^CS^α | CSEABart's disease | Positive | Positive |  | 🗸 |
| 45 | --^SEA^/αα | Alpha-thalassemia 1 heterozygote | Positive | Positive |  | 🗸 |
| 46 | β^IVSI-1^/β^E^, --^SEA^/αα | Beta-thalassemia/HbE disease with alpha-thalassemia 1 trait | Positive | Negative |  | 🗸 |
| 47 | -α^3.7^/αα | Alpha-thalassemia 2 heterozygote | Positive | Negative | 🗸 |  |
| 48 | --^SEA^/-α^3.7^ | HbH disease | Positive | Positive |  | 🗸 |
| 49 | --^SEA^/-α^3.7^ | HbH disease | Positive | Positive |  | 🗸 |
| 50 | β^4bp^/β^E^, --^SEA^/αα | Beta-thalassemia/HbE disease with alpha-thalassemia 1 trait | Positive | Positive |  | 🗸 |
| 51 | --^SEA^/-α^3.7^ | HbH disease | Positive | Positive |  | 🗸 |
| 52 | --^SEA^/αα | Alpha-thalassemia 1 heterozygote | Positive | Positive |  | 🗸 |
| 53 | --^SEA^/α^CS^α | HbH-CS disease | Positive | Positive |  | 🗸 |
| 54 | --^SEA^/αα | Alpha-thalassemia 1 heterozygote | Positive | Positive |  | 🗸 |
| 55 | αα/αα | Normal Hemoglobin type | Negative | Negative | 🗸 |  |
| 56 | --^SEA^/αα | Alpha-thalassemia 1 heterozygote | Positive | Positive |  | 🗸 |
| 57 | β^E^/β^N^, --^SEA^/αα | HbE heterozygote with alpha-thalassemia 1 trait | Positive | Positive |  | 🗸 |
| 58 | --^SEA^/αα | Alpha-thalassemia 1 heterozygote | Positive | Positive |  | 🗸 |
| 59 | --^SEA^/αα | Alpha-thalassemia 1 heterozygote | Positive | Positive |  | 🗸 |
| 60 | --^SEA^/-α^3.7^ | HbH disease | Positive | Positive |  | 🗸 |
| 61 | --^SEA^/-α^4.2^ | HbH disease | Positive | Positive |  | 🗸 |
| 62 | --^SEA^/αα | Alpha-thalassemia 1 heterozygote | Positive | Positive |  | 🗸 |
| 63 | --^SEA^/αα | Alpha-thalassemia 1 heterozygote | Positive | Positive |  | 🗸 |
| 64 | β^E^/β^E^, αα/αα | Homozygous HbE | Negative | Negative |  | 🗸 |
| 65 | β^E^/β^N^, αα/αα | HbE heterozygote | Negative | Negative | 🗸 |  |
| 66 | -α^3.7^/αα | Alpha-thalassemia 2 heterozygote | Negative | Negative | 🗸 |  |
| 67 | --^SEA^/αα | Alpha-thalassemia 1 heterozygote | Positive | Positive |  | 🗸 |
| 68 | --^SEA^/αα | Alpha-thalassemia 1 heterozygote | Positive | Positive |  | 🗸 |
| 69 | --^SEA^/αα | Alpha-thalassemia 1 heterozygote | Positive | Positive |  | 🗸 |
| 70 | β^E^/β^N^, αα/αα | HbE heterozygote | Negative | Negative | 🗸 |  |
| 71 | β^E^/β^N^, α^CS^α/αα | HbE heterozygote with Hb Constant Spring heterozygote | Negative | Positive | 🗸 |  |
| 72 | --^SEA^/αα | Alpha-thalassemia 1 heterozygote | Positive | Positive |  | 🗸 |
| 73 | -α^3.7^/αα | Alpha-thalassemia 2 heterozygote | Positive | Positive | 🗸 |  |
| 74 | β^E^/β^E^, αα/αα | Homozygous HbE | Negative | Positive |  | 🗸 |
| 75 | β^19^/β^N^, αα/αα | Beta-thalassemia heterozygote | Negative | Negative |  | 🗸 |
| 76 | β^E^/β^E^, αα/αα | Homozygous HbE | Negative | Negative |  | 🗸 |
| 77 | --^SEA^/α^CS^α | HbH-CS disease | Positive | Positive |  | 🗸 |
| 78 | --^SEA^/α^CS^α | HbH-CS disease | Positive | Positive |  | 🗸 |
| 79 | α^CS^α/αα | Hb Constant Spring heterozygote | Negative | Positive | 🗸 |  |
| 80 | β^E^/β^N^, --^SEA^/α^CS^α | CSEABart's disease | Positive | Positive |  | 🗸 |
| 81 | α^CS^α/αα | Hb Constant Spring heterozygote | Negative | Positive | 🗸 |  |
| 82 | β^E^/β^N^, --^SEA^/αα | HbE heterozygote with alpha-thalassemia 1 trait | Positive | Positive |  | 🗸 |
| 83 | --^SEA^/αα | Alpha-thalassemia 1 heterozygote | Positive | Positive |  | 🗸 |
| 84 | --^SEA^/αα | Alpha-thalassemia 1 heterozygote | Positive | Positive |  | 🗸 |
| 85 | αα/αα | Normal Hemoglobin type | Negative | Negative | 🗸 |  |
| 86 | --^SEA^/αα | Alpha-thalassemia 1 heterozygote | Positive | Positive |  | 🗸 |
| 87 | β^E^/β^N^, -α^3.7^/αα | HbE heterozygote with alpha-thalassemia 2 heterozygote | Negative | Negative | 🗸 |  |
| 88 | -α^3.7^/αα | Alpha-thalassemia 2 heterozygote | Positive | Negative | 🗸 |  |
| 89 | -α^3.7^/αα | Alpha-thalassemia 2 heterozygote | Negative | Positive | 🗸 |  |
| 90 | --^SEA^/αα | Alpha-thalassemia 1 heterozygote | Positive | Positive |  | 🗸 |
| 91 | --^SEA^/αα | Alpha-thalassemia 1 heterozygote | Positive | Positive |  | 🗸 |
| 92 | --^SEA^/αα | Alpha-thalassemia 1 heterozygote | Positive | Positive |  | 🗸 |
| 93 | -α^3.7^/αα | Alpha-thalassemia 2 heterozygote | Negative | Negative | 🗸 |  |
| 94 | β^E^/β^N^, --^SEA^/α^CS^α | CSEABart's disease | Positive | Positive |  | 🗸 |
| 95 | β^E^/β^N^, --^SEA^/αα | HbE heterozygote with alpha-thalassemia 1 trait | Positive | Negative |  | 🗸 |
| 96 | α^Int (-T)^α/αα | Alpha-thalassemia 2 heterozygote | Positive | Positive | 🗸 |  |
| 97 | --^SEA^/α^Int (-T)^α/αα | HbH disease | Positive | Positive |  | 🗸 |
| 98 | -α^3.7^/-α^3.7^ | Alpha-thalassemia 2 homozygote | Positive | Positive |  | 🗸 |
| 99 | -α^3.7^/αα | Alpha-thalassemia 2 heterozygote | Negative | Negative | 🗸 |  |
| 103 | αα/αα | Normal Hemoglobin type | Negative | Negative | 🗸 |  |
| 101 | αα/αα | Normal Hemoglobin type | Negative | Negative | 🗸 |  |
| 102 | β^E^/β^N^, αα/αα | HbE heterozygote | Negative | Negative | 🗸 |  |
| 103 | αα/αα | Normal Hemoglobin type | Negative | Negative | 🗸 |  |
| 104 | α^CS^α/αα | Hb Constant Spring heterozygote | Negative | Positive | 🗸 |  |
| 105 | αα/αα | Normal Hemoglobin type | Negative | Negative | 🗸 |  |
| 106 | β^E^/β^N^, --^SEA^/αα | HbE heterozygote with alpha-thalassemia 1 trait | Positive | Positive |  | 🗸 |
| 107 | --^SEA^/-α^3.7^ | HbH disease | Positive | Positive |  | 🗸 |
| 108 | αα/αα | Normal Hemoglobin type | Negative | Negative | 🗸 |  |
| 109 | --^SEA^/αα | Alpha-thalassemia 1 heterozygote | Positive | Positive |  | 🗸 |
| 110 | β^E^/β^N^, -α^3.7^/-α^3.7^ | HbE heterozygote with alpha-thalassemia 2 homozygote | Negative | Positive |  | 🗸 |
| 111 | αα/αα | Normal Hemoglobin type | Negative | Negative | 🗸 |  |
| 112 | αα/αα | Normal Hemoglobin type | Negative | Negative | 🗸 |  |
| 113 | αα/αα | Normal Hemoglobin type | Negative | Positive | 🗸 |  |
| 114 | αα/αα | Normal Hemoglobin type | Negative | Negative | 🗸 |  |
| 115 | αα/αα | Normal Hemoglobin type | Negative | Negative | 🗸 |  |
| 116 | αα/αα | Normal Hemoglobin type | Negative | Negative | 🗸 |  |
| 117 | αα/αα | Normal Hemoglobin type | Positive | Negative | 🗸 |  |
| 118 | αα/αα | Normal Hemoglobin type | Negative | Negative | 🗸 |  |
| 119 | αα/αα | Normal Hemoglobin type | Negative | Negative | 🗸 |  |
| 120 | αα/αα | Normal Hemoglobin type | Negative | Negative | 🗸 |  |
| 121 | αα/αα | Normal Hemoglobin type | Negative | Negative | 🗸 |  |
| 122 | αα/αα | Normal Hemoglobin type | Negative | Negative | 🗸 |  |
| 123 | αα/αα | Normal Hemoglobin type | Positive | Negative | 🗸 |  |
| 124 | αα/αα | Normal Hemoglobin type | Negative | Negative | 🗸 |  |
| 125 | αα/αα | Normal Hemoglobin type | Negative | Negative | 🗸 |  |
| 126 | αα/αα | Normal Hemoglobin type | Negative | Negative | 🗸 |  |
| 127 | αα/αα | Normal Hemoglobin type | Negative | Negative | 🗸 |  |
| 128 | αα/αα | Normal Hemoglobin type | Negative | Negative | 🗸 |  |
| 129 | αα/αα | Normal Hemoglobin type | Negative | Negative | 🗸 |  |
| 130 | αα/αα | Normal Hemoglobin type | Negative | Negative | 🗸 |  |
| 131 | αα/αα | Normal Hemoglobin type | Negative | Negative | 🗸 |  |
| 132 | αα/αα | Normal Hemoglobin type | Negative | Negative | 🗸 |  |
| 133 | αα/αα | Normal Hemoglobin type | Negative | Negative | 🗸 |  |
| 134 | αα/αα | Normal Hemoglobin type | Negative | Negative | 🗸 |  |
| 135 | αα/αα | Normal Hemoglobin type | Negative | Negative | 🗸 |  |
| 136 | αα/αα | Normal Hemoglobin type | Negative | Negative | 🗸 |  |
| 137 | αα/αα | Normal Hemoglobin type | Negative | Negative | 🗸 |  |
| 138 | αα/αα | Normal Hemoglobin type | Negative | Negative | 🗸 |  |
| 139 | αα/αα | Normal Hemoglobin type | Positive | Negative | 🗸 |  |
| 140 | αα/αα | Normal Hemoglobin type | Negative | Negative | 🗸 |  |
| 141 | αα/αα | Normal Hemoglobin type | Negative | Negative | 🗸 |  |
| 142 | αα/αα | Normal Hemoglobin type | Negative | Negative | 🗸 |  |
| 143 | αα/αα | Normal Hemoglobin type | Negative | Negative | 🗸 |  |
| 144 | --^SEA^/αα | Alpha-thalassemia 1 heterozygote | Positive | Positive |  | 🗸 |
| 145 | --^SEA^/αα | Alpha-thalassemia 1 heterozygote | Positive | Positive |  | 🗸 |
| 146 | -α^3.7^/-α^4.2^ | Alpha-thalassemia 2 homozygote | Negative | Positive |  | 🗸 |
| 147 | -α^3.7^/αα | Alpha-thalassemia 2 heterozygote | Negative | Negative | 🗸 |  |
| 148 | αα/αα | Normal Hemoglobin type | Negative | Negative | 🗸 |  |
| 149 | β^E^/β^N^, --^SEA^/-α^3.7^ | EABart's disease | Positive | Positive |  | 🗸 |
| 150 | β^E^/β^N^, --^SEA^/-α^T^ | EABart's disease | Positive | Negative |  | 🗸 |
| 151 | αα/αα | Normal Hemoglobin type | Positive | Negative | 🗸 |  |
| 152 | --^SEA^/αα | Alpha-thalassemia 1 heterozygote | Positive | Positive |  | 🗸 |
| 153 | αα/αα | Normal Hemoglobin type | Negative | Negative | 🗸 |  |
| 154 | αα/αα | Normal Hemoglobin type | Positive | Negative | 🗸 |  |
| 155 | --^SEA^/αα | Alpha-thalassemia 1 heterozygote | Positive | Positive |  | 🗸 |
| 156 | --^SEA^/αα | Alpha-thalassemia 1 heterozygote | Positive | Positive |  | 🗸 |
| 157 | -α^3.7^/-α^3.7^ | Alpha-thalassemia 2 homozygote | Negative | Positive |  | 🗸 |
| 158 | -α^3.7^/αα | Alpha-thalassemia 2 heterozygote | Positive | Positive | 🗸 |  |
| 159 | α^CS^α/αα | Hb Constant Spring heterozygote | Negative | Positive | 🗸 |  |
| 160 | α^CS^α/αα | Hb Constant Spring heterozygote | Positive | Positive | 🗸 |  |
| 161 | β^E^/β^N^, -α^3.7^/αα | HbE heterozygote with alpha-thalassemia 2 heterozygote | Positive | Negative | 🗸 |  |
| 162 | β^E^/β^N^, --^SEA^/α^CS^α | CSEABart's disease | Positive | Positive |  | 🗸 |
| 163 | β^E^/β^E^, αα/αα | Homozygous HbE | Positive | Negative |  | 🗸 |
| 164 | β^E^/β^N^, αα/αα | HbE heterozygote | Positive | Negative | 🗸 |  |
| 165 | β^E^/β^N^, αα/αα | HbE heterozygote | Negative | Positive | 🗸 |  |
| 166 | α^CS^α/α^CS^α | Homozygous Constant Spring | Negative | Positive | 🗸 |  |
| 167 | α^CS^α/αα | Hb Constant Spring heterozygote | Negative | Positive | 🗸 |  |
| 168 | β^E^/β^N^, αα/αα | HbE heterozygote | Negative | Negative | 🗸 |  |
| 169 | β^4bp^/β^N^, -α^3.7^/αα | Beta-thalassemia heterozygote with alpha-thalassemia 2 trait | Negative | Negative |  | 🗸 |
| 170 | -α^3.7^/-α^3.7^ | Alpha-thalassemia 2 homozygote | Positive | Positive |  | 🗸 |
| 171 | -α^3.7^/αα | Alpha-thalassemia 2 heterozygote | Negative | Positive | 🗸 |  |
| 172 | --^SEA^/αα | Alpha-thalassemia 1 heterozygote | Positive | Positive |  | 🗸 |
| 173 | --^SEA^/αα | Alpha-thalassemia 1 heterozygote | Positive | Positive |  | 🗸 |
| 174 | αα/αα | Normal Hemoglobin type | Negative | Negative | 🗸 |  |
| 175 | --^SEA^/αα | Alpha-thalassemia 1 heterozygote | Positive | Positive |  | 🗸 |
| 176 | αα/αα | Normal Hemoglobin type | Negative | Negative | 🗸 |  |
| 177 | αα/αα | Normal Hemoglobin type | Negative | Negative | 🗸 |  |
| 178 | --^SEA^/αα | Alpha-thalassemia 1 heterozygote | Positive | Positive |  | 🗸 |
| 179 | β^E^/β^N^, -α^3.7^/-α^3.7^ | HbE heterozygote with alpha-thalassemia 2 homozygote | Negative | Positive |  | 🗸 |
| 180 | -α^3.7^/αα | Alpha-thalassemia 2 heterozygote | Negative | Negative | 🗸 |  |
| 181 | -α^3.7^/-α^3.7^ | Alpha-thalassemia 2 homozygote | Positive | Positive |  | 🗸 |
| 182 | -α^3.7^/α^CS^α | Alpha-thalassemia 2 heterozygote with Hb CS heterozygote | Positive | Positive | 🗸 |  |
| 183 | --^SEA^/αα | Alpha-thalassemia 1 heterozygote | Positive | Positive |  | 🗸 |
| 184 | -α^3.7^/-α^4.2^ | Alpha-thalassemia 2 homozygote | Negative | Positive |  | 🗸 |
| 185 | -α^3.7^/αα | Alpha-thalassemia 2 heterozygote | Positive | Positive | 🗸 |  |
| 186 | --^SEA^/αα | Alpha-thalassemia 1 heterozygote | Positive | Positive |  | 🗸 |
| 187 | αα/αα | Normal Hemoglobin type | Negative | Negative | 🗸 |  |
| 188 | -α^4.2^/αα | Alpha-thalassemia 2 heterozygote | Negative | Negative | 🗸 |  |
| 189 | β^E^/β^N^, αα/αα | HbE heterozygote | Positive | Negative | 🗸 |  |
| 190 | β^E^/β^N^, -α^3.7^/αα | HbE heterozygote with alpha-thalassemia 2 heterozygote | Negative | Negative | 🗸 |  |
| 191 | -α^3.7^/αα | Alpha-thalassemia 2 heterozygote | Negative | Positive | 🗸 |  |
| 192 | α^CS^α/αα | Hb Constant Spring heterozygote | Negative | Positive | 🗸 |  |
| 193 | --^SEA^/αα | Alpha-thalassemia 1 heterozygote | Positive | Positive |  | 🗸 |
| 194 | β^E^/β^N^, αα/αα | HbE heterozygote | Negative | Positive | 🗸 |  |
| 195 | β^E^/β^E^, αα/αα | Homozygous HbE | Negative | Negative |  | 🗸 |

* Alpha-thalassemia 1 heterozygote (α^0^-thalassemia heterozygote); Alpha-thalassemia 2 heterozygote (α^+^-thalassemia heterozygote)

** High = MCV ≥ 80 fL and MCH ≥ 27 pg (High MCV/MCH); Low = MCV < 80 fL or MCH < 27 pg (Low MCV/MCH)

One hundred ninety-five subjects with various thalassemia and normal hemoglobin were recruited in this study. The genotypes and phenotypes of each sample are shown. Samples with MCV ≥ 80 fL and MCH ≥ 27 pg (High MCV/MCH) or MCV < 80 fL or MCH < 27 pg (Low MCV/MCH) are indicated. The IC strip test results (+, positive; -, negative) of each subject are also indicated.
